# Diagnostic implications of renin reactivity in confirmatory tests: a comparative study of direct renin concentration and plasma renin activity in primary aldosteronism

**DOI:** 10.1530/EC-25-0302

**Published:** 2025-10-28

**Authors:** Tomoyuki Nagasaka, Yoshiaki Hishida, Hiroki Yasuda, Shuhei Kusuda, Nanami Ikeda, Shutaro Uchiyama, Yui Kubo, Mayuko Kano, Tomoko Nakagawa, Yuta Nakamura, Shiko Asai, Kenichi Yokota, Keiko Yanagisawa, Masakatsu Sone

**Affiliations:** Division of Metabolism and Endocrinology, Department of Internal Medicine, St. Marianna University School of Medicine, Kawasaki, Japan

**Keywords:** primary aldosteronism, renin responsiveness, direct renin concentration, plasma renin activity

## Abstract

**Graphical Abstract:**

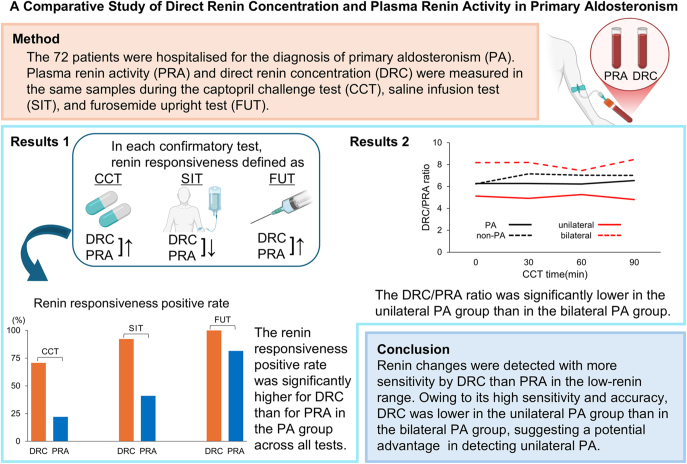

**Abstract:**

**Objective**

Renin measurement is key to diagnosing primary aldosteronism (PA). However, it remains unclear whether plasma renin activity (PRA) or direct renin concentration (DRC), which are based on different principles, offers superior performance. This study aimed to compare their diagnostic potential in PA.

**Methods**

This cross-sectional observational study was conducted at a single institution and enrolled consecutive patients hospitalised for PA diagnosis. PRA and DRC were measured for the same samples during the captopril challenge, saline infusion, and furosemide upright tests. We evaluated renin responsiveness, defined as renin changes from baseline in each test, and the relationship between PRA and DRC.

**Results**

Seventy-two patients were classified into PA and non-PA groups. The patients in the PA group were further classified into unilateral and bilateral PA groups using adrenal vein sampling. Renin responsiveness positivity was significantly higher for DRC than for PRA in the PA group across all tests. The DRC/PRA ratio did not differ significantly between PA and non-PA groups, while the DRC/PRA ratio was significantly lower in the unilateral PA group than in the bilateral PA group. The correlation between PRA and DRC was weak in the PA group, especially in the low-renin range.

**Conclusions**

Renin changes were more consistently detected with DRC than with PRA in the low-renin range. The DRC/PRA ratio was lower in unilateral PA than in bilateral PA, suggesting potential utility for recognising unilateral PA, although this finding should be considered exploratory.

## Introduction

Primary aldosteronism (PA), a major cause of secondary hypertension, is endocrinologically characterised by autonomous aldosterone oversecretion and renin suppression (1). Clinically, patients with PA exhibit a significantly higher incidence of cardiovascular events and renal complications than those with essential hypertension and comparable blood pressure levels (2, 3, 4). However, specific treatments, including surgical and pharmacological interventions, can reduce PA-related cardiovascular risk, highlighting the importance of early diagnosis and timely intervention (5).

The aldosterone-to-renin ratio (ARR) is a key parameter involved in PA screening. Plasma aldosterone concentration (PAC) has been used to measure aldosterone levels, and renin has been assessed using two distinct methods: plasma renin activity (PRA) and direct renin concentration (DRC). The ARR calculated using PRA is denoted as ‘ARR-P’, and that with DRC is denoted as ‘ARR-D’. In this study, the units of ARR-P and ARR-D were ng/dL per ng/mL/h and ng/dL per pg/mL, respectively, and are omitted hereafter for readability.

The renin enzymatic activity estimated by PRA is an indirect method involving measurement of the rate of angiotensin I produced using angiotensinogen as the substrate per unit time. In contrast, the renin concentration measured by DRC is a direct method, excluding prorenin and using a two-site sandwich immunoassay with specific monoclonal antibodies (6, 7). Although PRA and DRC are correlated, they fundamentally represent different aspects of the renin system. Previous studies have characterised the differences between PRA and DRC, highlighting their measurement principles, clinical applications, and limitations (8).

PRA has a relatively high lower limit of quantification (LLOQ; 0.1–0.2 ng/mL/h), posing challenges in the detection of the low-renin levels characteristics of PA (9, 10). In cases of severe PA with marked renin suppression, PRA, the denominator in ARR-P calculations, often reaches its LLOQ and remains unchanged. Thus, in these cases, ARR-P depends solely on the numerator, PAC. This limitation of PRA has been recognised and reported previously (9, 10). Moreover, early studies, including the PAPY study, reported discrepancies in the correlation between PRA and DRC before and after the captopril challenge test (CCT), attributing these discrepancies to potential inaccuracies in DRC assays at low-renin levels (11). However, subsequent advancements have led to highly sensitive DRC assays capable of detecting lower concentrations with greater precision, thereby enhancing their utility in PA diagnosis (7).

Conventionally, PRA has been used as the standard method for renin measurement (6, 7, 10, 11). However, a recent international survey by Naruse *et al.* reported that DRC surpassed PRA as the more commonly used assay in clinical practice (12). Accordingly, no definitive conclusion has been reached regarding whether PRA or DRC is more useful for renin measurement during PA diagnosis (9). In particular, the differences and correlation changes during confirmatory testing, a key diagnostic step, remain insufficiently studied.

Therefore, we performed a comparative analysis of PRA and DRC in the same sample at baseline and during confirmatory testing to determine their diagnostic utility for PA.

## Materials and methods

### Study design

This single-centre, cross-sectional observational study was conducted at St. Marianna University Hospital (Kawasaki, Japan) between April 2022 and September 2024. The study protocol was approved by the Clinical Research Ethics Committee of St. Marianna University School of Medicine (Approval No. 6109, 4 July 2023) and adhered to the revised Declaration of Helsinki. Informed consent was obtained from all patients. Clinical and biochemical data were extracted from the hospital’s electronic medical records. This manuscript was prepared in accordance with the Strengthening the Reporting of Observational Studies in Epidemiology (STROBE) guidelines (13).

Consecutive patients suspected of having PA, based on the Japan Endocrine Society screening criteria (14), and those who underwent confirmatory testing during hospitalisation in the study period were included in the study.

The inclusion criteria were as follows: i) male or female patients aged ≥18 years; ii) positive PA screening (ARR-P > 10 and PAC >6 ng/dL); and iii) confirmatory testing performed during hospitalisation.

The exclusion criteria were as follows: i) failure to complete any confirmatory test; ii) prior diagnosis of PA; iii) absence of informed consent; and iv) considered ineligible for other reasons at the discretion of the principal investigator. The exclusion criterion iv) was included to account for cases in which patients were deemed ineligible due to comorbid conditions that could potentially affect renin or aldosterone levels, such as liver cirrhosis or nephrotic syndrome, or for other unforeseen reasons.

### Hormone evaluation and assays

Hormone assessments were performed after switching medications that affect the renin-angiotensin system (RAS), including angiotensin II receptor blockers, angiotensin-converting enzyme inhibitors, beta-blockers, and diuretics, to calcium channel blockers (amlodipine or nifedipine) or α-blockers (doxazosin) at least 3 weeks before hospital admission. Mineralocorticoid receptor antagonists were switched to these medications at least 6 weeks before admission. Oral potassium supplementation was provided for patients with hypokalaemia.

Hormone levels were measured using commercially available kits. Blood samples were collected using EDTA-2K tubes for all assays. The time from blood collection to centrifugation was within 15 min for all samples. If the measured values were below the LLOQ of the assay, the LLOQ was assigned as the measured value.

PAC was measured using the Lumipulse Presto Aldosterone assay (CLEIA, Fujirebio Inc., Japan). Blood samples were centrifuged at 4–8°C to separate plasma and stored at −80°C until analysis. The assay had a measurement range of 0.4–200.0 ng/dL. The coefficients of variation (CVs) were 0.4, 0.4, and 0.5% at low, medium, and high concentrations (9.38, 75.33, and 128.65 ng/dL, respectively), and 10% at the lowest quality control (QC) level (0.2465 ng/dL).

PRA was measured using the Yamasa Renin Activity Kit (EIA, YAMASA CORPORATION, Japan), following the same plasma preparation protocol as that used for PAC. No protease inhibitors were used in this assay. Blood samples were centrifuged at 4–8°C to separate plasma and stored at −80°C until analysis. The assay had a measurement range of 0.2–45 ng/mL/h. The CVs were 3.4, 3.5, and 1.7% at low, medium, and high concentrations (1.6, 4.7, and 9.6 ng/mL/h, respectively), and 11% at the lowest evaluated concentration (0.41 ng/mL/h) (15).

DRC was measured using the Lumipulse Presto Renin kit (CLEIA, Fujirebio Inc., Japan). The renin concentration measured using this kit is referred to as active renin concentration (ARC). However, as ARC corresponds to what is commonly referred to as DRC in many published studies, the term DRC is used consistently throughout this manuscript for clarity (1, 11, 16). Unlike PAC and PRA, blood samples for DRC were centrifuged at room temperature (approximately 24°C) before plasma separation and stored at −80°C. The assay had a measurement range of 0.20–1,000.0 pg/mL. The CVs were 0.5, 0.6, and 0.7% at low, medium, and high concentrations (21.09, 416.37, and 711.39 pg/mL, respectively), and 10% at the lowest QC level (0.0929 pg/mL). For example, the CV was 4.9% at a DRC level of 0.17 pg/mL. DRC can be expressed in mU/L by multiplying the value calculated in pg/mL by 1.67 (11).

### Diagnosis of PA, confirmatory testing, and subtype classification

PA was diagnosed based on the diagnostic criteria of the Japan Endocrine Society (14). Confirmatory testing included CCT, saline infusion test (SIT), and furosemide upright test (FUT). CCT involved oral administration of 50 mg captopril followed by blood sample collection every 30 min up to 90 min, with a positive result defined as an ARR-P of ≥20 at 60 or 90 min. SIT was performed in the seated position and involved 2 L saline infusion over 240 min, with blood samples collected before and after the infusion; a PAC of ≥6.0 ng/dL at 240 min was considered positive. FUT involved intravenous administration of 40 mg furosemide, followed by patients remaining in an upright position for 120 min, with blood samples collected every 30 min up to 120 min, and a PRA of <2.0 ng/mL/h at 120 min was considered positive.

PA subtype classification was based on adrenal venous sampling (AVS) in patients who provided informed consent and were deemed eligible by their physicians. Blood samples were obtained from both adrenal veins and the inferior vena cava (IVC) before and after 20 min of adrenocorticotropic hormone (ACTH) stimulation via 250 μg rapid intravenous tetracosactide. According to the Japan Endocrine Society guidelines, successful cannulation was defined as an adrenal vein-to-IVC cortisol ratio of ≥2 before and ≥5 after ACTH stimulation (14, 17, 18). Lateralisation was assessed only after successful cannulation. Unilateral PA was diagnosed if the post-ACTH aldosterone-to-cortisol ratio of one adrenal vein relative to the contralateral side was >4, while bilateral PA was diagnosed if the ratio was ≤4 (14, 17).

### Classification of the patients

Patients were categorised into those testing positive in PA screening but negative in all confirmatory tests (non-PA group) and those with at least one positive confirmatory test leading to a PA diagnosis (PA group). Within the PA group, patients who underwent AVS and were diagnosed with unilateral aldosterone hypersecretion were stratified into the unilateral PA group, while those with bilateral secretion were classified into the bilateral PA group.

### Performed analyses

The utility of PRA and DRC for PA screening and confirmatory testing was compared using three analyses. First, renin responsiveness, defined as changes in renin levels during confirmatory tests, was estimated to compare the potential of PRA or DRC to reflect these variations. Confirmatory tests were categorised as renin stimulation tests (CCT and FUT) when renin levels increased following drug administration, or renin suppression tests (SIT) when renin secretion decreased due to saline infusion. Renin responsiveness was defined as:

- CCT: PRA or DRC at 60 or 90 min exceeding the baseline after 50 mg captopril administration.
- SIT: PRA or DRC at 240 min lower than baseline after a 2 L saline infusion.
- FUT: PRA or DRC at 120 min exceeding baseline after 40 mg intravenous furosemide administration.

Renin responsiveness was compared between the measurement methods (PRA vs DRC) and across patient groups.

Second, the changes in the correlation between PRA and DRC during confirmatory testing were assessed by analysing the DRC/PRA ratio at each blood sampling time point within each test and comparing it across groups.

Third, the general correlation between PRA and DRC was assessed by plotting and comparing the values at baseline and pre-test measurements between the PA and non-PA groups. To further explore the utility of PRA and DRC in the most representative confirmatory test, the diagnostic performance of ARR-P and ARR-D in detecting unilateral PA was compared in the CCT.

### Statistical analysis

Statistical analyses were performed using JMP Pro 16 (SAS Institute Inc., USA) and R version 4.5.0 (The R Foundation for Statistical Computing, Austria). The normality of the data was assessed using the Shapiro–Wilk test. Results are presented as means ± standard deviations (SDs) for normally distributed data or as medians (interquartile ranges (IQRs)) for non-normally distributed data. Differences between the two groups were analysed using Student’s *t*-test for normally distributed data or the Mann–Whitney U test for non-normally distributed data. The distribution of categorical variables was assessed using the chi-squared test. Positive rates of renin responsiveness between DRC and PRA were compared using a one-sided binomial test, with the observed rate of one method used as the expected value under the null hypothesis. Spearman’s rank correlation and least-squares regression analyses were used to evaluate the correlations between two variables. Logistic regression analysis was performed using variables that showed significant differences between the PA and non-PA groups or between the unilateral and bilateral PA groups. Receiver operating characteristic (ROC) curve analysis was conducted to determine the optimal cut-off values using the Youden index. The DeLong test was used to compare ROC curves derived from diagnostic indices involving PRA and DRC. As sensitivity analyses, two additional evaluations were conducted: one in which a stricter threshold for renin responsiveness was defined as a change exceeding twice the CV of each assay (DRC and PRA), and another in which PRA values below the LLOQ were imputed as half the LLOQ, considering that a substantial proportion of PRA measurements was expected to fall below the quantification limit. Statistical significance was set at a *P*-value of <0.05.

## Results

**Figure 1:**
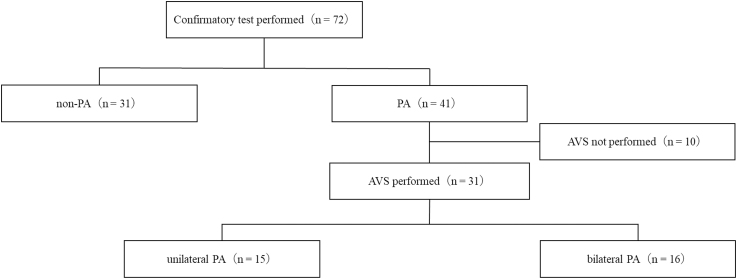
Participant recruitment flowchart. Abbreviations: AVS, adrenal venous sampling; PA, primary aldosteronism.

The patient recruitment flowchart is presented in Fig. 1. Of the 72 patients who underwent confirmatory tests, 41 were diagnosed with PA. Eight patients had their antihypertensive medications affecting the RAS switched to appropriate alternatives before hospital admission. The baseline characteristics of the PA and non-PA groups are summarised in Table 1, and those of the unilateral and bilateral PA groups are presented in Table 2.

**Table 1:** Baseline characteristics of the primary aldosteronism and non-primary aldosteronism groups.

	PA (*n* = 41)	Non-PA (*n* = 31)	*P*
Female, *n* (%)	21 (51.2)	24 (77.4)	0.023*
Age, years	51.9 ± 11.0	56.8 ± 12.0	0.079
BMI, kg/m^2^	24.7 ± 3.4	23.0 ± 3.5	0.051
SBP, mmHg	141.4 ± 17.7	128.7 ± 13.0	0.002*
DBP, mmHg	93.7 ± 13.0	83.9 ± 11.6	0.002*
Serum albumin, mg/dL	4.3 ± 0.3	4.3 ± 0.4	0.878
eGFR, mL/min/1.73 m^2^	76.8 ± 18.8	73.6 ± 12.2	0.416
Serum sodium, mEq/L	140.5 ± 1.7	140.5 ± 1.9	0.993
Serum potassium, mEq/L	3.8 ± 0.3	3.9 ± 0.3	0.065
Urinary aldosterone, μg/day	10.7 ± 6.1	6.6 ± 4.1	0.007*
Urinary sodium, mEq/day	112.3 ± 48.5	107.0 ± 49.4	0.695
Urinary potassium, mEq/day	38.7 ± 16.1	33.2 ± 12.8	0.175
PAC, ng/dL	9.5 (6.7–13.2)	7.0 (5.5–9.2)	0.010*
PRA, ng/mL/h	0.2 (0.2–0.3)	0.2 (0.2–0.4)	0.080
DRC, pg/mL	1.1 (0.8–2.0)	1.9 (1.4–2.5)	0.200
ARR-P, ng/dL/ng/mL per h	37.7 (26.7–59.0)	26.0 (15.8–37.9)	0.002*
ARR-D, ng/dL/pg/mL	7.5 (4.2–15.5)	3.9 (2.3–4.8)	0.010*

Data are expressed as means ± standard deviations or medians (interquartile ranges). Comparisons between the two groups were performed using Student’s *t*-test for normally distributed variables, the Mann–Whitney U test for non-normally distributed variables, and the chi-squared test for categorical variables. eGFR was calculated using the equation established for the Japanese population by the Japanese Society of Nephrology (19): eGFR (mL/min/1.73 m^2^) = 194 × serum creatinine^−1^·^094^ × age^−0^·^287^ (×0.739 for female patients).

* *P* < 0.05.

Abbreviations: ARR-D, plasma aldosterone concentration/direct renin concentration; ARR-P, plasma aldosterone concentration/plasma renin activity; BMI, body mass index; BW, body weight; DBP, diastolic blood pressure; DRC, direct renin concentration; eGFR, estimated glomerular filtration rate; PA, primary aldosteronism; PAC, plasma aldosterone concentration; PRA, plasma renin activity; SBP, systolic blood pressure.

**Table 2:** Baseline characteristics of the unilateral and bilateral primary aldosteronism groups.

	Unilateral PA (*n* = 15)	Bilateral PA (*n* = 16)	*P*
Female, *n* (%)	5 (33.3)	7 (43.8)	0.567
Age, years	51.5 ± 11.1	48.8 ± 5.9	0.403
BMI, kg/m^2^	24.5 ± 3.8	25.5 ± 3.3	0.446
SBP, mmHg	137.5 ± 14.5	148.9 ± 18.3	0.075
DBP, mmHg	92.4 ± 13.5	96.9 ± 12.1	0.350
Serum albumin, mg/dL	4.3 ± 0.3	4.4 ± 0.2	0.142
eGFR, mL/min/1.73 m^2^	85.1 ± 20.4	75.7 ± 15.4	0.173
Serum sodium, mEq/L	141.3 ± 1.5	140.2 ± 1.6	0.058
Serum potassium, mEq/L	3.5 ± 0.2	3.8 ± 0.2	0.002*
Urinary aldosterone, μg/day	15.6 ± 8.4	9.1 ± 2.3	0.013*
Urinary sodium, mEq/day	129.4 ± 49.8	118.1 ± 45.2	0.610
Urinary potassium, mEq/day	48.5 ± 25.2	36.3 ± 9.7	0.140
PAC, ng/dL	12.0 (9.7–15.2)	8.7 (6.2–9.9)	0.007*
PRA, ng/mL/h	0.2 (0.2–0.4)	0.2 (0.2–0.3)	0.981
DRC, pg/mL	0.9 (0.8–1.3)	1.0 (0.8–0.9)	1.000
ARR-P, ng/dL/ng/mL per h	57.5 (32.5–68.3)	31.3 (17.2–44.3)	0.066
ARR-D, ng/dL/pg/mL	15.2 (7.0–18.2)	8.4 (5.4–12.9)	0.269

Data are expressed as means ± standard deviations or medians (interquartile ranges). Comparisons between the two groups were performed using Student’s *t*-test for normally distributed variables and the Mann–Whitney U test for non-normally distributed variables, and the chi-squared test for categorical variables. eGFR was calculated using the equation established for the Japanese population by the Japanese Society of Nephrology (19): eGFR (mL/min/1.73 m^2^) = 194 × serum creatinine^−1^·^094^ × age^−0^·^287^ (×0.739 for female patients).

* *P* < 0.05.

Abbreviations: ARR-D, plasma aldosterone concentration/direct renin concentration; ARR-P, plasma aldosterone concentration/plasma renin activity; BMI, body mass index; BW, body weight; DBP, diastolic blood pressure; DRC, direct renin concentration; eGFR, estimated glomerular filtration rate; PA, primary aldosteronism; PAC, plasma aldosterone concentration; PRA, plasma renin activity; SBP, systolic blood pressure.

Among the 41 patients diagnosed with PA, 31 underwent AVS for lateralisation, wherein 15 were diagnosed with unilateral PA and 16 with bilateral PA. No patients met the exclusion criteria.

For confirmatory tests (*n* = 72), CCT was performed in 71 patients (98.6%). SIT was conducted in 68 patients (94.4%), excluding those for whom SIT was deemed inappropriate owing to age or comorbidities. FUT was performed in 51 patients (70.8%); however, one patient with missing DRC data was excluded. In some of the remaining 50 patients, FUT was discontinued owing to discomfort. The number of patients evaluated in FUT at each time point was 49 at 30 min, 44 at 60 min, 40 at 90 min, and 36 at 120 min.

### Evaluation of renin responsiveness in each confirmatory test

**Figure 2:**
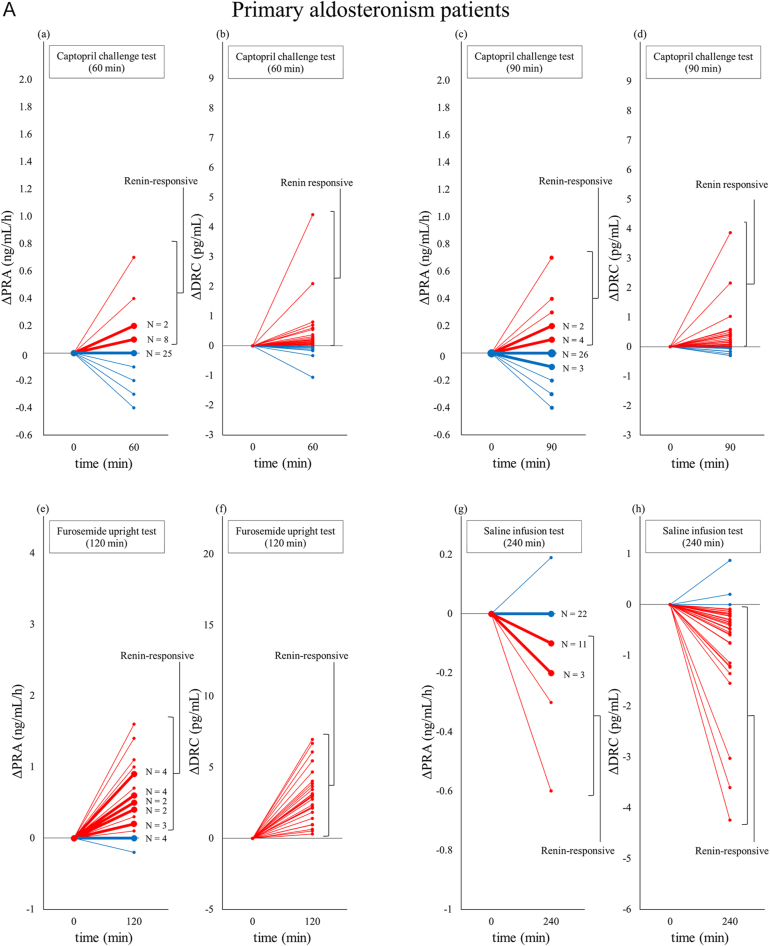

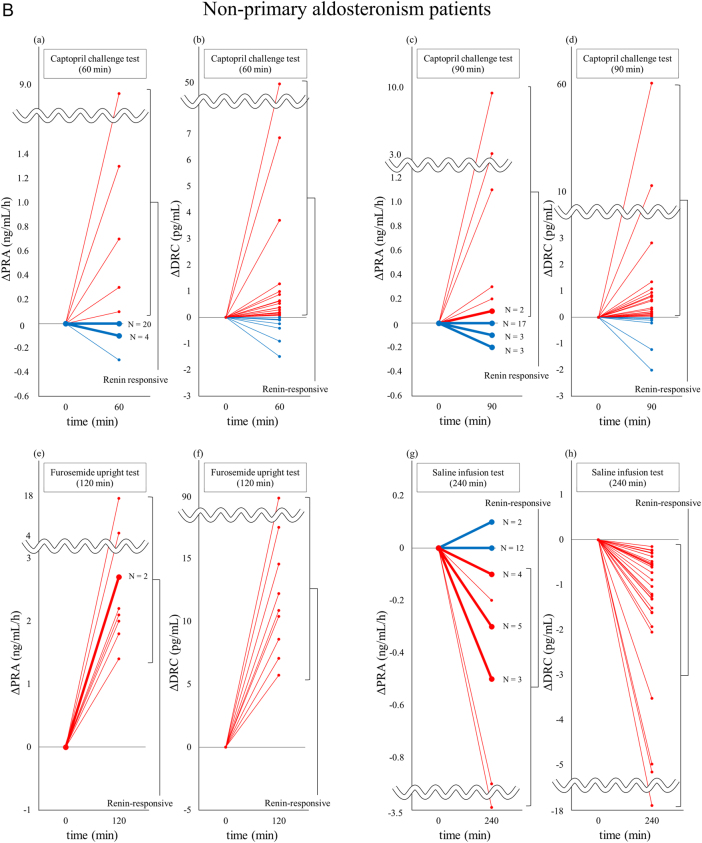
(A) Renin changes in confirmatory tests measured using two different assay principles in the primary aldosteronism group. Abbreviations: DRC, direct renin concentration; PRA, plasma renin activity. In the CCT, cases with increased renin levels after captopril administration were considered renin-responsive. (a) and (c) Changes in PRA (ΔPRA) from baseline to 60 and 90 min in CCT. The number of cases with the exact change is indicated by N in the figure, with line thickness corresponding to the frequency. (b) and (d) Changes in DRC (ΔDRC) from baseline to 60 and 90 min in CCT. No identical changes were observed. In the SIT, cases with renin levels decreasing after saline infusion were considered renin-responsive. (e) Changes in PRA (ΔPRA) from baseline to 240 min in SIT. The number of cases with the exact change is indicated by N in the figure, with line thickness corresponding to the frequency. (f) Changes in DRC (ΔDRC) from baseline to 240 min in SIT. No identical changes were observed. In the furosemide upright test (FUT), patients with increased renin levels after furosemide administration were considered renin-responsive. (g) Changes in PRA (ΔPRA) from baseline to 120 min in FUT. The number of cases with the exact change is indicated by N in the figure, with line thickness corresponding to the frequency. (h) Changes in DRC (ΔDRC) from baseline to 120 min in FUT. No identical changes were observed. (B) Renin changes in confirmatory tests measured using two different assay principles in the non-primary aldosteronism group. Abbreviations: DRC, direct renin concentration; PRA, plasma renin activity. In the CCT, cases with increased renin levels after captopril administration were considered renin-responsive. (a) and (c) Changes in PRA (ΔPRA) from baseline to 60 and 90 min in CCT. The number of cases with the exact change is indicated by N in the figure, with line thickness corresponding to the frequency. (b) and (d) Changes in DRC (ΔDRC) from baseline to 60 and 90 min in CCT. No identical changes were observed. In the SIT, cases with renin levels decreasing after saline infusion were considered renin-responsive. (e) Changes in PRA (ΔPRA) from baseline to 240 min in SIT. The number of cases with the exact change is indicated by N in the figure, with line thickness corresponding to the frequency. (f) Changes in DRC (ΔDRC) from baseline to 240 min in SIT. No identical changes were observed. In the furosemide upright test (FUT), patients with increased renin levels after furosemide administration were considered renin-responsive. (g) Changes in PRA (ΔPRA) from baseline to 120 min in FUT. The number of cases with the exact change is indicated by N in the figure, with line thickness corresponding to the frequency. (h) Changes in DRC (ΔDRC) from baseline to 120 min in FUT. No identical changes were observed.

Renin responsiveness was assessed in the PA and non-PA groups, and the results are summarised in Table 3 and Fig. 2A and B.

**Table 3:** Renin responsiveness at each sampling time point in each confirmatory test.

Test		DRC, *n* (%)	PRA, *n* (%)	*P*
**PA group**				
Captopril challenge	30 min	32 (80.0)	10 (24.4)	<0.001*
	60 min	30 (75.0)	11 (26.8)	<0.001*
	90 min	28 (70.0)	9 (22.0)	<0.001*
Saline infusion	240 min	36 (92.3)	16 (41.0)	<0.001*
Furosemide upright	30 min	31 (100)	19 (61.3)	<0.001*
	60 min	29 (100)	19 (65.5)	<0.001*
	90 min	28 (100)	23 (82.1)	0.004*
	120 min	27 (100)	22 (81.5)	0.004*
**Non-** **PA** ** group**				
Captopril challenge	30 min	20 (66.7)	7 (23.3)	<0.001*
	60 min	21 (70.0)	5 (16.7)	<0.001*
	90 min	23 (76.7)	7 (23.3)	<0.001*
Saline infusion	240 min	29 (100)	15 (51.7)	<0.001*
Furosemide upright	30 min	18 (100)	16 (88.9)	0.120
	60 min	15 (100)	13 (86.7)	0.117
	90 min	12 (100)	10 (83.3)	0.112
	120 min	9 (100)	9 (100)	1.000

Values are *n* (%). Positive rates of renin responsiveness between DRC and PRA were compared using a one-sided binomial test, with the observed rate of one method used as the expected value under the null hypothesis.

* *P* < 0.05.

Abbreviations: DRC, direct renin concentration; PRA, plasma renin activity.

### PA group

During CCT at 60 min, renin responsiveness was observed in 26.8% of the patients (11/41) using PRA and 75.0% of them (30/40) using DRC, with significantly greater responsiveness for DRC (*P* < 0.001). At 90 min, responsiveness was observed in 22.0% (9/41) of the patients using PRA and in 70.0% (28/40) of the patients using DRC (*P* < 0.001). During SIT, at 240 min, responsiveness was observed in 41.0% (16/39) of the patients using PRA and 92.3% (36/39) of the patients using DRC, with a significantly higher detection rate with DRC (*P* < 0.001). During FUT, at 120 min, responsiveness was observed in 81.5% (22/27) of the patients using PRA and in 100% of the patients (27/27) using DRC, with a significantly higher detection rate with DRC (*P* = 0.004).

### Non-PA group

During CCT at 60 min, responsiveness was observed in 16.7% (5/30) of the patients using PRA and 70.0% of the patients (21/30) using DRC, with significantly higher responsiveness with DRC (*P* < 0.001). At 90 min, responsiveness was observed in 23.3% (7/30) of the patients using PRA and in 76.6% (23/30) of the patients using DRC (*P* < 0.001). During SIT, responsiveness was observed in 51.7% (15/29) of the patients using PRA and in 100% (29/29) of the patients using DRC at 240 min, with a significantly higher detection rate with DRC (*P* < 0.001). During FUT, at 120 min, all nine cases (100%) exhibited responsiveness using both PRA and DRC, with no significant difference between the two parameters (*P* = 1.000).

Overall, in many cases, PRA remained unchanged from baseline, even after loading. This lack of change was due to the large minimum change unit in the assay, causing multiple cases to exhibit identical values, resulting in overlapping data points along a single line (Fig. 2A(a, c, e, g) and B(a, c, e, g)). The specific numbers of patients with PRA and DRC measurements below the LLOQ at each time point of the confirmatory tests in each group are presented in Supplementary Table S1 (see section on Supplementary materials given at the end of the article). Notably, no patients had DRC values below the LLOQ at any time point, whereas the number of patients with PRA values below the LLOQ was higher in the PA group than in the non-PA group.

In addition, several sensitivity analyses were performed to ensure the robustness of the findings. Because some PRA values reached LLOQ, a sensitivity analysis was performed; cases with PRA at the LLOQ were assigned a value corresponding to half the LLOQ (i.e. 0.1 ng/mL/h) to evaluate renin responsiveness. Even with this adjustment, DRC consistently demonstrated significantly better detection of renin responsiveness compared to PRA (Supplementary Table S2).

A similar sensitivity analysis was conducted to address the concern that changes in PRA and DRC during confirmatory testing might not be biologically meaningful when considering the CV. Renin responsiveness was defined as a change exceeding twice the CV of the respective assay. The lowest PRA and DRC values in this study were 0.2 ng/mL/h and 0.28 pg/mL, with corresponding CVs of 11 and 4.9%, respectively, according to the assay specifications. Thus, thresholds of ≥20% for PRA and ≥10% for DRC were used to define renin responsiveness in this analysis. This sensitivity analysis also yielded consistent results, with renin responsiveness observed significantly more frequently with DRC than with PRA (Supplementary Table S3).

Furthermore, multivariable logistic regression was performed using DRC-based renin responsiveness in each confirmatory test as the dependent variable. As no variables were significant in the univariable analysis, factors with high potential for confounding were included in the model, followed by a stepwise backward elimination procedure. This analysis did not identify any parameters that significantly confounded renin responsiveness (Supplementary Table S4).

### Changes in the DRC/PRA ratio across confirmatory tests

**Figure 3:**
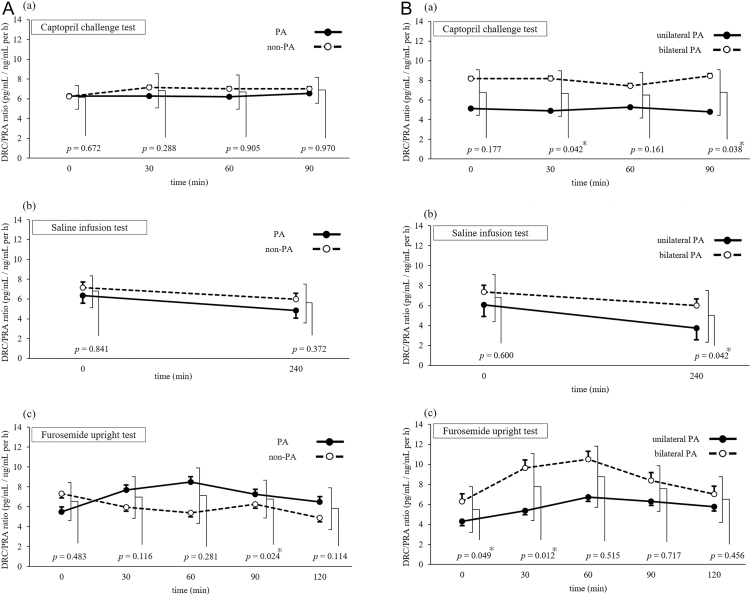
(A) DRC/PRA ratio across confirmatory tests in the primary and non-primary aldosteronism groups. Abbreviations: DRC, direct renin concentration; PA, primary aldosteronism; PRA, plasma renin activity. Solid and dashed lines represent PA and non-PA groups, respectively. Error bars indicate standard error. (A) CCT. (B) SIT. (C) Furosemide upright test. Differences between the two groups were analysed using Student’s *t*-test. *P < 0.05. (B) DRC/PRA ratio across confirmatory tests in the unilateral and bilateral primary aldosteronism groups. Abbreviations: DRC, direct renin concentration; PA, primary aldosteronism; PRA, plasma renin activity. Solid and dashed lines represent unilateral and bilateral PA groups, respectively. Error bars indicate standard error. (A) CCT. (B) SIT. (C) Furosemide upright test. Differences between the two groups were analysed using Student’s *t*-test. *P < 0.05.

Initially, the DRC/PRA ratio was compared between the PA and non-PA groups (Fig. 3A). In FUT, a significant difference was observed only at 90 min, with a lower ratio in the non-PA group than in the PA group (*P* = 0.024). No significant differences were observed for other time points with FUT, CCT, or SIT.

Next, the DRC/PRA ratio was analysed among the PA subtypes. Overall, across all confirmatory tests, the unilateral PA group had a lower DRC/PRA ratio than the bilateral PA group (Fig. 3B). Specifically, the DRC/PRA ratio was significantly lower in the unilateral PA group at 30 min (*P* = 0.042) and 90 min (*P* = 0.038) in CCT, at 240 min in SIT (*P* = 0.042), and at baseline (*P* = 0.049) and 30 min (*P* = 0.012) in FUT.

Finally, as an exploratory analysis, the diagnostic performance of the DRC/PRA ratio at 90 min in CCT (the time point with the lowest *P*-value) was evaluated for identifying unilateral PA, which yielded an AUC of 0.72 (Supplementary Fig. S2).

### General correlation between PRA and DRC

**Figure 4:**
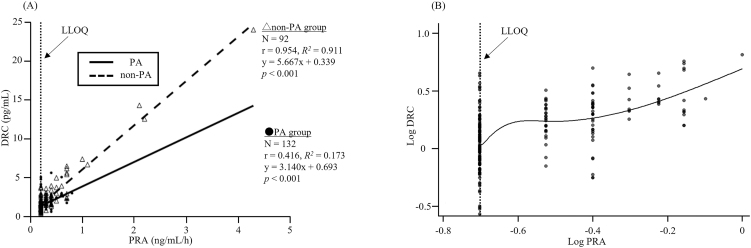
Scatter plots of PRA and DRC. Abbreviations: DRC, direct renin concentration; LLOQ, lower limit of quantification; PA, primary aldosteronism; PRA, plasma renin activity. (A) Least-squares regression analysis of PRA and DRC at baseline and pre-test measurements in each group. Solid lines represent the PA group, and dashed lines represent the non-PA group. The vertical dashed line represents the LLOQ for PRA (0.2 ng/mL/h). (B) Scatter plot of log-transformed PRA and DRC at baseline and pre-test measurements in all patients with a smoothing spline curve. The logarithm is to the base 10. The vertical dashed line represents the LLOQ for log PRA (approximately −0.7).

PRA and DRC at baseline and pre-test measurements across all patients had a significant correlation (*r* = 0.901, *P* < 0.001) with a linear regression equation of DRC = 0.17 + 5.56 × PRA. In the non-PA group, a significant correlation was observed between baseline PRA and DRC (*r* = 0.954, *R^2^* = 0.911, *P* < 0.001), with a linear regression equation of DRC = 0.34 + 5.67 × PRA (Fig. 4A). In the PA group, PRA and DRC also had a positive correlation; however, the correlation coefficient was notably lower than that in the non-PA group (*r* = 0.416, *R^2^* = 0.173, *P* < 0.001) with a regression equation of DRC = 0.69 + 3.14 × PRA (Fig. 4A). In addition, in the PA group with strong renin suppression, the correlation coefficient in the linear regression analysis was low, and the values had high variability. Therefore, an additional exploratory analysis was conducted using the logarithmic transformation of PRA and DRC for all the PA samples. The results revealed that in the low-renin range, the slope of the smoothed spline curve varied more markedly (Fig. 4B).

To compare the utility of PRA and DRC in confirmatory testing, the diagnostic performance of ARR-P and ARR-D for detecting unilateral PA was evaluated in CCT, which was the most representative test with the fewest missing values. The results are presented in Supplementary Fig. S1. At baseline (pre-captopril), the AUCs for ARR-P and ARR-D were 0.820 and 0.835, respectively (*P* = 0.719). At 60 min post-administration, the corresponding AUCs were 0.775 and 0.813 (*P* = 0.518), and at 90 min, 0.746 and 0.767 (*P* = 0.766). While no significant differences were observed at any time point, the AUCs for ARR-D tended to be higher than those for ARR-P.

## Discussion

This study evaluated the relationship between PRA and DRC and compared their utility in PA diagnosis. Specifically, both parameters were measured in the same samples at baseline and during confirmatory testing. Intergroup differences were also assessed between the PA and non-PA groups and among the PA subtypes classified based on AVS into unilateral and bilateral PA groups.

This study conducted three key analyses. First, renin responsiveness was evaluated in each confirmatory test, highlighting the differences in the diagnostic characteristics of PRA and DRC. Second, the DRC/PRA ratio at each sampling time point across the loading tests was analysed, providing a detailed assessment of the relationship between PRA and DRC in each group. Finally, scatter plots were used to examine the overall correlation between the DRC and PRA values, emphasising their distribution and relationship.

### Renin responsiveness: PRA vs DRC

The first key analysis focused on the difference in renin responsiveness between PRA and DRC in confirmatory tests.

In the PA group, DRC had significantly greater sensitivity than PRA in detecting both positive and negative renin responses, likely due to differences in the assay characteristics at low-renin levels. PRA fell below LLOQ in 46% (196/423) of the baseline samples and had a large minimum change unit (±0.1 ng/mL/hr), making subtle renin activity changes difficult to detect. In contrast, DRC remained within the measurable range for all samples with a smaller minimum change unit (±0.01 pg/mL), facilitating the detection of finer variations in renin concentration. DRC had superior sensitivity in detecting renin responses in confirmatory tests. As reported by Taki *et al.* (10), PRA, measured in 0.1 ng/mL/h increments, functions as a discrete variable, while DRC, measured in 0.01 pg/mL increments, functions as a continuous variable with enhanced sensitivity at lower concentrations. This characteristic was evident in the confirmatory tests, with no change in PRA values in many cases before and after loading, leading to overlapping data points along a single line (Fig. 2A(a, c, e, g)).

In the non-PA group, DRC also detected renin responsiveness significantly more frequently than PRA in the CCT and SIT. However, in FUT, unlike in the PA group, PRA detected renin responsiveness at a level comparable to that of DRC. This discrepancy likely resulted from differences in renin suppression between the PA and non-PA groups. Renin suppression was milder in the non-PA group, likely allowing PRA to have sufficient changes beyond its minimum change unit in response to furosemide stimulation, thereby enabling the detection of renin responsiveness at a level similar to that of DRC.

Among the three confirmatory tests in this study, SIT was used to evaluate renin suppression, whereas both FUT and CCT were used to evaluate renin stimulation. Between the two renin measurement methods, DRC and PRA, which differ in their approaches for assessing renin, the method with greater responsiveness to confirmatory-test loading would be more suitable for diagnosis.

In the multivariable logistic regression analysis, no parameters emerged as significant confounders for the presence or absence of renin responsiveness in any of the confirmatory tests (Supplementary Table S4). This finding suggests that DRC-based renin responsiveness can be evaluated regardless of PA status (PA vs non-PA) or lateralisation subtype (unilateral vs bilateral PA). Owing to its superior measurement sensitivity in the low-renin range, DRC allows a more precise evaluation of changes in renin even under marked renin suppression, which may have implications for future clinical assessment of PA.

### Evaluation of the DRC/PRA ratio across groups

The second key analysis focused on the relationship between PRA and DRC, assessed using the DRC/PRA ratio in each confirmatory test.

The DRC/PRA ratio was first compared between the PA and non-PA groups, and no significant differences were observed at most sampling points (Fig. 3A). The only exception was at 90 min in FUT, with a significantly higher DRC/PRA ratio in the PA group than in the non-PA group. However, this result lacked consistency and may have been influenced by multiple comparisons.

Next, the DRC/PRA ratio was analysed in the PA subtypes (unilateral vs bilateral PA). Across most sampling points, the unilateral PA group had a lower DRC/PRA ratio than the bilateral PA group, with statistically significant differences at several time points (Fig. 3B). The PAPY study reported changes in the PRA–DRC relationship in the low-renin range (11). As unilateral PA primarily comprises aldosterone-producing adenomas (APAs), this group has stronger renin suppression and lower renin levels than bilateral PA, likely explaining the difference in DRC/PRA ratios.

As an additional analysis, the diagnostic performance of the DRC/PRA ratio at 90 min during CCT was evaluated for identifying unilateral PA. The resulting AUC was 0.72, indicating that the DRC/PRA ratio may have limited utility for PA lateralisation (Supplementary Fig. S2). However, the lower DRC/PRA ratio in the unilateral PA group suggests that ARR-D was higher than ARR-P in this group. Thus, the DRC-based ARR evaluation may better detect more severe forms of PA, including APA.

### Evaluation of the general correlation between PRA and DRC

The third key analysis concerned the overall correlation between PRA and DRC at baseline and was assessed using scatter plots. This study observed a positive correlation between PRA and DRC in all patients, with DRC being approximately 3–6 times greater than PRA. Previous studies have reported a DRC/PRA ratio of 4–7 (6, 7, 10, 20), indicating no major discrepancy between our findings and previous reports.

Next, we separately analysed the correlation between the PA and non-PA groups (Fig. 4A). The Pearson’s correlation coefficient was lower in the PA group than in the non-PA group. Furthermore, the multiple *R^2^* value was lower, suggesting that the proportion of the total variance explained by the linear regression model was smaller in the PA group than in the non-PA group. As this finding was attributable to lower renin levels in the PA group based on scatter plots, logarithmic transformation and smoothing spline analyses were performed for all patients. These analyses revealed a distortion in the correlation between PRA and DRC in the low-renin range.

These results are consistent with those of previous studies (10, 11) reporting a weakened correlation between PRA and DRC under low-renin conditions. In this study, the observed change in the correlation may be attributed to PRA behaving as a discrete variable in the low-renin range.

Furthermore, in an exploratory comparison of the diagnostic performance of ARR-P and ARR-D during CCT to evaluate the utility of PRA and DRC in confirmatory testing, no significant differences in AUCs were observed at any time points. However, ARR-D consistently showed a trend toward higher AUCs than ARR-P, suggesting that DRC is not inferior to PRA in this setting.

### Implications of the findings

These three key analyses collectively highlight the advantages of DRC over PRA for PA diagnosis. DRC had greater utility than PRA in three key aspects, including enabling a more precise evaluation of hormone dynamics in PA diagnosis, serving as a more robust indicator of the unilateral PA subtype, and providing more reliable renin measurement levels. These advantages may be attributed to the higher precision of DRC in measuring low concentrations and its smaller minimum change unit compared to that of PRA. Our results are consistent with those of the PAPY study (11), which reported a shift in the correlation between PRA and DRC in the low- and high-renin ranges. However, as DRC assays have improved over time and now facilitate more accurate measurements at lower concentrations, DRC can detect subtle renin fluctuations more sensitively than PRA.

Recent studies have suggested that DRC is more suitable than PRA for evaluating PA in patients with PRA levels ≤2.0 ng/mL/h (10). Moreover, a recent international survey conducted across 33 institutions in 17 countries reported that approximately 60% of the facilities adopted DRC for renin measurement (12). Owing to the improved accuracy of DRC in reflecting *in vivo* renin dynamics, our findings support the transition from PRA to DRC for renin measurements in PA diagnostics.

### Limitations and future perspectives

Despite its advantages, the clinical application of DRC remains limited owing to the lack of established cut-off values corresponding to PRA-based PA screening thresholds (10). Further investigations are needed to define the appropriate cut-off values and optimise the clinical application of DRC. This study comprehensively evaluates the relationship between PRA and DRC from multiple perspectives. At a time when the number of institutions adopting DRC-based renin assays is increasing, it may serve as a bridge facilitating the transition from PRA-based to DRC-based renin assessment.

Furthermore, the high sensitivity of DRC in the low-renin range demonstrated in this study may contribute to improving the accuracy of various confirmatory tests. For example, it could increase the clinical relevance of CCT – which imposes relatively less burden on patients among renin-stimulation tests – while reducing the necessity for the more burdensome FUT. This strategy, in turn, may not only alleviate patient burden but also enhance cost-effectiveness.

This study has some limitations. It was conducted at a single institution with relatively small sample sizes, likely limiting the generalisability of the findings. In addition, multiple comparison corrections were not applied owing to the sample size constraints. Thus, multiple comparisons in large sample sizes should be considered in future studies. Notably, the non-PA group comprised patients with positive screening results who were not diagnosed with PA in confirmatory tests and therefore does not reflect the general population with essential hypertension. Finally, as PRA is the predominant renin measurement in Japan and has traditionally been employed at our institution, selection bias favouring individuals with lower PRA values during PA screening cannot be excluded.

## Conclusion

Due to its higher sensitivity in the low-renin range, DRC provides complementary information to PRA in the diagnosis and management of PA. While ARR-D tended to be higher than ARR-P in unilateral PA compared with bilateral PA, this observation requires validation in larger cohorts. It should currently be regarded as hypothesis-generating rather than definitive for subtype classification.

## Supplementary materials



## Declaration of interest

The authors declare that they have no known competing financial interests or personal relationships that could be construed to influence the work reported in this paper.

## Funding

This study was partly supported by a Grant-in-Aid from the Ministry of Health, Labour, and Welfare, Japan (No. 23FC1041 for research on intractable adrenal disorders).

## Data availability

Anonymised data and analytical code used in this study are available from the corresponding author upon reasonable request.

## References

[1] Vaidya A, Hundemer GL, Nanba K, et al. Primary aldosteronism: state-of-the-art review. Am J Hypertens 2022 35 967–988. (10.1093/ajh/hpac079) 35767459 PMC9729786

[2] Monticone S, D’Ascenzo F, Moretti C, et al. Cardiovascular events and target organ damage in primary aldosteronism compared with essential hypertension: a systematic review and meta-analysis. Lancet Diabetes Endocrinol 2018 6 41–50. (10.1016/S2213-8587(17)30319-4) 29129575

[3] Ohno Y, Sone M, Inagaki N, et al. Prevalence of cardiovascular disease and its risk factors in primary aldosteronism: a multicenter study in Japan. Hypertension 2018 71 530–537. (10.1161/HYPERTENSIONAHA.117.10263) 29358460

[4] Kawashima A, Sone M, Inagaki N, et al. Renal impairment is closely associated with plasma aldosterone concentration in patients with primary aldosteronism. Eur J Endocrinol 2019 181 339–350. (10.1530/EJE-19-0047) 31319380

[5] Catena C, Colussi G, Nadalini E, et al. Cardiovascular outcomes in patients with primary aldosteronism after treatment. Arch Intern Med 2008 168 80–85. (10.1001/archinternmed.2007.33) 18195199

[6] Teruyama K, Naruse M, Tsuiki M, et al. Novel chemiluminescent immunoassay to measure plasma aldosterone and plasma active renin concentrations for the diagnosis of primary aldosteronism. J Hum Hypertens 2022 36 77–85. (10.1038/s41371-020-00465-5) 33564064 PMC8766281

[7] Morimoto R, Ono Y, Tezuka Y, et al. Rapid screening of primary aldosteronism by a novel chemiluminescent immunoassay. Hypertension 2017 70 334–341. (10.1161/HYPERTENSIONAHA.117.09078) 28652474 PMC5613948

[8] Kao TW, Chen JY, Liu JH, et al. Diagnostic efficacy of aldosterone-to-renin ratio to screen primary aldosteronism in hypertension: a systematic review and meta-analysis. Ther Adv Endocrinol Metab 2024 15 20420188241303429. (10.1177/20420188241303429) 39669529 PMC11635879

[9] Unger N, Lopez Schmidt I, Pitt C, et al. Comparison of active renin concentration and plasma renin activity for the diagnosis of primary hyperaldosteronism in patients with an adrenal mass. Eur J Endocrinol 2004 150 517–523. (10.1530/eje.0.1500517) 15080782

[10] Taki Y, Kono T, Teruyama K, et al. Comparative analysis of aldosterone and renin assays for primary aldosteronism screening. Sci Rep 2024 14 26040. (10.1038/s41598-024-75645-1) 39472614 PMC11522277

[11] Rossi GP, Barisa M, Belfiore A, et al. The aldosterone-renin ratio based on the plasma renin activity and the direct renin assay for diagnosing aldosterone-producing adenoma. J Hypertens 2010 28 1892–1899. (10.1097/HJH.0b013e32833d2192) 20683340

[12] Naruse M, Murakami M, Katabami T, et al. International multicenter survey on screening and confirmatory testing in primary aldosteronism. Eur J Endocrinol 2023 188 lvac002. (10.1093/ejendo/lvac002) 36726325

[13] von EE, Altman DG, Egger M, et al. The strengthening the reporting of observational studies in epidemiology (STROBE) statement: guidelines for reporting observational studies. Lancet 2007 370 1453–1457. (10.1016/S0140-6736(07)61602-X) 18064739

[14] Naruse M, Katabami T, Shibata H, et al. Japan endocrine society clinical practice guideline for the diagnosis and management of primary aldosteronism 2021. Endocr J 2022 69 327–359. (10.1507/endocrj.EJ21-0508) 35418526

[15] Uzu T, Nakamura T, Ashida N, et al. Development and basic evaluation of the renin activity kit “Yamasa” using the EIA method [in Japanese]. Igaku To Yakugaku 2016 73 311.

[16] Funder JW, Carey RM, Mantero F, et al. The management of primary aldosteronism: case detection, diagnosis, and treatment: an endocrine society clinical practice guideline. J Clin Endocrinol Metab 2016 101 1889–1916. (10.1210/jc.2015-4061) 26934393

[17] Rossi GP, Auchus RJ, Brown M, et al. An expert consensus statement on use of adrenal vein sampling for the subtyping of primary aldosteronism. Hypertension 2014 63 151–160. (10.1161/HYPERTENSIONAHA.113.02097) 24218436

[18] Webb R, Mathur A, Chang R, et al. What is the best criterion for the interpretation of adrenal vein sample results in patients with primary hyperaldosteronism? Ann Surg Oncol 2012 19 1881–1886. (10.1245/s10434-011-2121-5) 22048631 PMC4034057

[19] Matsuo S, Imai E, Horio M, et al. Revised equations for estimated GFR from serum creatinine in Japan. Am J Kidney Dis 2009 53 982–992. (10.1053/j.ajkd.2008.12.034) 19339088

[20] Umemura S, Arima H, Arima S, et al. The Japanese society of hypertension guidelines for the management of hypertension (JSH 2019). Hypertens Res 2019 42 1235–1281. (10.1038/s41440-019-0284-9) 31375757

