# Supplementary material for: Diagnostic implications of renin reactivity in confirmatory tests: a comparative study of direct renin concentration and plasma renin activity in primary aldosteronism
Source: Endocr Connect. 2025 Oct 28;14(10):e250302. doi: 10.1530/EC-25-0302 (PMC12569981; doi:10.1530/EC-25-0302)
Supplement: Supplementary file 1 [file supplementary_materials.pdf]

Table S1. Number of Patients with Plasma Renin Activity or Direct Renin Concentration at the lower limit of quantification in Each Confirmatory Test

	PA		non-PA	
	DRC	PRA	DRC	PRA
Number at LLOQ in CCT, n (%)				
0 min	0 (0.0)	26 (63.4)	0 (0.0)	6 (19.4)
30 min	0 (0.0)	23 (56.1)	0 (0.0)	7 (22.6)
60 min	0 (0.0)	20 (48.8)	0 (0.0)	9 (29.0)
90 min	0 (0.0)	22 (53.7)	0 (0.0)	8 (25.8)
Number at LLOQ in SIT, n (%)				
0 min	0 (0.0)	16 (41.0)	0 (0.0)	5 (17.2)
240 min	0 (0.0)	24 (61.5)	0 (0.0)	8 (27.6)
Number at LLOQ in FUT, n (%)				
0 min	0 (0.0)	19 (59.4)	0 (0.0)	4 (22.2)
30 min	0 (0.0)	8 (25.8)	0 (0.0)	1 (5.6)
60 min	0 (0.0)	8 (27.6)	0 (0.0)	0 (0.0)
90 min	0 (0.0)	4 (14.3)	0 (0.0)	0 (0.0)
120 min	0 (0.0)	3 (11.1)	0 (0.0)	0 (0.0)

Abbreviations: CCT, captopril challenge test; DRC, direct renin concentration; FUT, furosemide upright test; LLOQ, lower limit of quantification; PA, primary aldosteronism; PRA, plasma renin activity; SIT, saline infusion test.

Table S2. Renin Responsiveness at Each Sampling Time Point in Each Confirmatory Test (Sensitivity Analysis Using Half the LLOQ for PRA)

PA group

Test		DRC, n (%)	PRA, n (%)	*p*
Captopril challenge	30 min	32 (80.0)	11 (26.8)	< 0.001*
	60 min	30 (75.0)	12 (29.3)	< 0.001*
	90 min	28 (70.0)	12 (29.3)	< 0.001*
Saline infusion	240 min	36 (92.3)	19 (48.7)	< 0.001*
Furosemide upright	30 min	31 (100)	22 (71.0)	0.001*
	60 min	29 (100)	19 (65.5)	< 0.001*
	90 min	28 (100)	23 (82.1)	0.004*
	120 min	27 (100)	23 (85.2)	0.013*

Non-PA group

Test		DRC, n (%)	PRA, n (%)	*p*
Captopril challenge	30 min	20 (66.7)	8 (26.6)	< 0.001*
	60 min	21 (70.0)	5 (16.7)	< 0.001*

	90 min	23 (76.7)	8 (26.7)	< 0.001*
Saline infusion	240 min	29 (100)	15 (51.7)	< 0.001*
Furosemide upright	30 min	18 (100)	16 (88.9)	0.120
	60 min	15 (100)	13 (86.7)	0.117
	90 min	12 (100)	10 (83.3)	0.112
	120 min	9 (100)	9 (100)	1.000

The present sensitivity analysis was conducted using 0.1 ng/mL/h, which corresponds to half the LLOQ for PRA. Values are n (%). Positive rates of renin responsiveness between DRC and PRA were compared using a one-sided binomial test, with the observed rate of one method used as the expected value under the null hypothesis.

*$p < 0.05$.

Abbreviations: DRC, direct renin concentration; LLOQ, lower limit of quantification; PRA, plasma renin activity

Table S3. Renin Responsiveness at Each Sampling Time Point in Each Confirmatory Test (Sensitivity Analysis Using a ±2×CV Change as the Threshold)

PA group

Test		DRC, n (%)	PRA, n (%)	*p*
Captopril challenge	30 min	21 (52.5)	10 (24.4)	< 0.001*
	60 min	17 (75.0)	11 (26.8)	< 0.023*
	90 min	19 (47.5)	9 (22.0)	< 0.001*
Saline infusion	240 min	34 (87.2)	16 (41.0)	< 0.001*
Furosemide upright	30 min	31 (100)	19 (61.3)	< 0.001*
	60 min	29 (100)	19 (65.5)	< 0.001*
	90 min	28 (100)	23 (82.1)	0.004*
	120 min	27 (100)	22 (81.5)	0.004*

Non-PA group

Test		DRC, n (%)	PRA, n (%)	*p*
Captopril challenge	30 min	14 (46.7)	7 (23.3)	0.004*
	60 min	16 (53.3)	5 (16.7)	< 0.001*

	90 min	18 (60.0)	7 (23.3)	< 0.001*
Saline infusion	240 min	27 (93.1)	15 (51.7)	< 0.001*
Furosemide upright	30 min	17 (94.4)	16 (88.9)	0.390
	60 min	15 (100)	13 (86.7)	0.117
	90 min	12 (100)	10 (83.3)	0.112
	120 min	9 (100)	9 (100)	1.000

The present sensitivity analysis was conducted by defining renin responsiveness as a change exceeding twice the CV for each assay, namely ±10% for DRC and ±20% for PRA, in each confirmatory test. Values are n (%). Positive rates of renin responsiveness between DRC and PRA were compared using a one-sided binomial test, with the observed rate of one method used as the expected value under the null hypothesis.

*$p < 0.05$

Abbreviations: DRC, direct renin concentration; CV, coefficient of variation; LLOQ, lower limit of quantification; PRA, plasma renin activity.

Table S4. Logistic Regression Analysis for Renin Responsiveness at 90 Minutes in the Captopril Challenge Test

Variable	Odds Ratio	95% CI	*p*
Female. n (%)	2.070	0.449–9.570	0.351
SBP, mmHg	1.010	0.958–1.060	0.721
DBP, mmHg	1.030	0.964–1.100	0.385
Serum sodium, mEq/L	0.965	0.607–1.540	0.881
Serum potassium, mEq/L	2.370	0.167–33.800	0.524
Urinary potassium, mEq/day	1.000	0.940–1.070	0.932
Urinary aldosterone, µg/day	0.991	0.808–1.220	0.931
PAC, ng/dL	1.000	0.991–1.010	0.946
DRC at 0 min (pg/mL)	1.270	0.780–2.050	0.339

Logistic regression analysis was performed incorporating the variables listed in the table, followed by a stepwise backward elimination procedure. No variables were statistically significant.

*$p < 0.05$.

Abbreviations: DBP, diastolic blood pressure; DRC, direct renin concentration; PAC, plasma aldosterone concentration; PRA, plasma renin activity; SBP, systolic blood pressure.

Figure S1. Receiver Operating Characteristic Curves for PRA- and DRC-Based ARR in the Detection of Unilateral Primary Aldosteronism During the Captopril Challenge Test

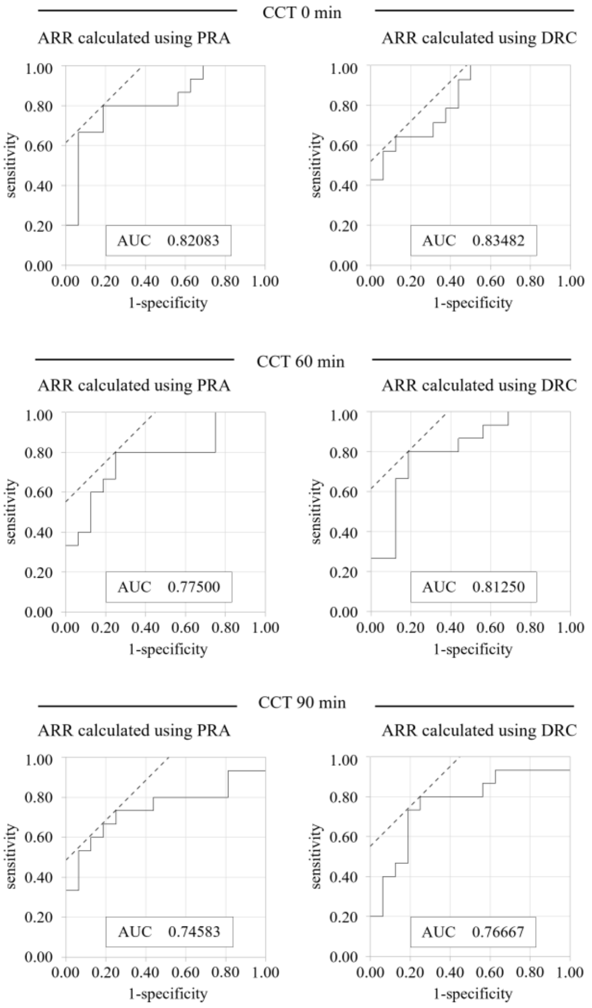

The dashed line indicates the line tangent to the ROC curve at the point corresponding to the maximum Youden index.

At CCT 0 min, the sensitivity and specificity were 0.800/0.812 in the left panel (ARR cutoff: 407.0) and 0.643/0.875 in the right panel (ARR cutoff: 96.6), respectively.

At CCT 60 min, the corresponding values were 0.800/0.750 (ARR cutoff: 245.0) and 0.800/0.812 (ARR cutoff: 43.3).

At CCT 90 min, they were 0.733/0.750 (ARR cutoff: 317.5) and 0.800/0.750 (ARR cutoff: 47.1), respectively.

Abbreviations: ARR, aldosterone-to-renin ratio; AUC, area under the curve; CCT, captopril challenge test; DRC, direct renin concentration; PRA, plasma renin activity.

Figure S2. Receiver Operating Characteristic Curve of the DRC/PRA Ratio After Captopril Administration in the Detection of Unilateral Primary Aldosteronism

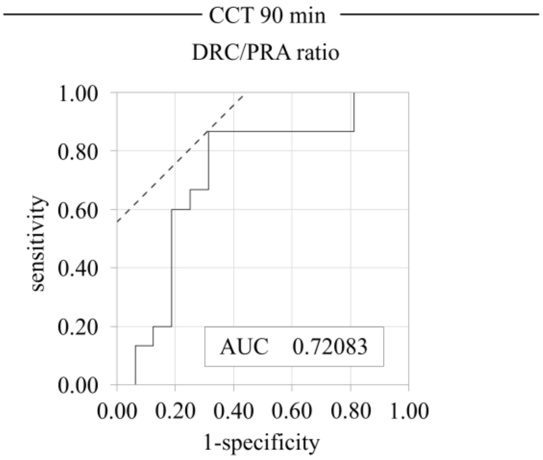

The dashed line indicates the line tangent to the ROC curve at the point corresponding to the maximum Youden index. The sensitivity and specificity at this point were 0.867 and 0.688, respectively (DRC/PRA ratio cutoff: 5.350).

Abbreviations: AUC, area under the curve; CCT, captopril challenge test; DRC, direct renin concentration; PRA, plasma renin activity.
